# Evaluation of soil and water conservation function in the Wugong mountain meadow based on the comprehensive index method

**DOI:** 10.1016/j.heliyon.2022.e11867

**Published:** 2022-11-28

**Authors:** Sohel Rana, Xuna Cheng, Yanfang Wu, Chuanwei Hu, Razia Sultana Jemim, Zhen Liu, Yanmei Wang, Qifei Cai, Xiaodong Geng, Xiaomin Guo, Zhi Li

**Affiliations:** a College of Forestry, Henan Agricultural University, Zhengzhou 450046, China; b Qianying Township Government, Pingdingshan 467493, China; c Palm Eco-Town Development Co., Ltd., Zhengzhou 450000, China; d College of Life Sciences, Henan Agricultural University, Zhengzhou 450046, China; e College of Forestry, Jiangxi Agricultural University, Nanchang 330045, China

**Keywords:** Wugong mountain, High altitude, Meadow soil, Soil and water conservation, Soil hydro-physiology

## Abstract

Wugong Mountain meadow landscape is well-known both at home and abroad because of its ornamental value. Our study aimed to comprehensively evaluate the function of soil and water conservation at different altitudes of Wugong Mountain meadow soil. The hydro-physical characteristics, including the soil bulk density, porosity, water content, water holding capacity, and permeability of meadow soil at 1600 m–1900 m altitudes, were analyzed. The results showed that the mountain meadow soil's hydro-physiological characteristics and water conservation function significantly differed with altitude. However, the trend of each index did not follow the same law with altitude change. There was a decrease in bulk density of the soil from 1700 m to 1900 m, but a significant increase in porosity and water-holding capacity. Despite the higher porosity and water holding capacity found at 1600 m than at 1700 m and 1800 m, a similar bulk density was found at 1600 m as 1700 m. In addition, the bulk density in the 0–20 cm layer was lower than that in the 20–40 cm layer, while the porosity and water-holding capacity were higher. A higher sequence of soil water conservation capacity was found in soil layers 0–20 cm depth at 1900, 1600, 1800, and 1700 m; in soil layers 20–40 cm depth, it was at 1900, 1800, 1700, and 1600 m. The study found that the sequence of the comprehensive performance of soil water conservation function was at 1900, 1600, 1800, and 1700 m altitudes in the Wugong mountain meadow area. Our comprehensive study of soil water conservation capacity provides a theoretical basis for the rational use of mountain meadow resources in subtropical regions.

## 1 Introduction

Soil water is essential for the survival of vegetation and other life forms. The ability of water conservation functions not only determines the quality of the living environment but also affects the material exchange and transmission process between the earth structure of interface (He et al., 2016; Li et al., 2015). Moreover, soil erosion is a global environmental problem. It can lead to soil structure destruction and nutrient loss, causing the degradation of soil functions (Morgan, 1995). Soil erosion also affects the hydrological process and cycling of crucial elements, such as carbon and nitrogen (Lal, 1999; Quinton et al., 2010).

The altitude is one of the key factors that affect the change of the ecological environment concerning climatic conditions. It can change the water, heat, light, etc., and then with identical climatic conditions, the soil properties will change and thus affect the growth of vegetation (Coners et al., 2016; Ragettli et al., 2014). The altitude profoundly affects the soil's inherent fertility and runoff-erosion behavior (Bowman et al., 2002; Mani, 1990). Many soil fertility characteristics (including organic matter content, pH, cation exchange capacity, phosphate sorption, and phosphorus availability) show significant altitudinal variations (Jobbagy and Jackson, 2000). In different areas, soil and water conservation techniques are widely used to reduce water and soil loss through engineering, tillage, and biological measures. For example, the effects of soil and water conservation forests on conserving soil and water are reflected mainly in alleviating surface runoff scour and maintaining or recovering soil fertility (Wang et al., 2016). In addition, researchers compared different conservation techniques in China's purple soil hilly region (Liu et al., 2008) and observed the soil and water conservation efficiency in the Rocky Mountains of Southwest China (Sun et al., 2017). Many scholars have applied different methods in different areas, including the Universal Soil Erosion Equation (USLE) (Fu et al., 2011; Wischmeier et al., 1978), Revised Universal Soil Loss Equation (RUSLE) (Kulikov et al., 2020), Water Erosion Prediction Project (WEPP) (Zheng et al., 2020), Erosion Productivity Impact Calculator (EPIC), and Areal Nonpoint Source Watershed Environment Response Simulation (ANSWERS) (Bhuyan et al., 2002). Some scholars have predicted of model for the transport of nitrates in soils (Ekeleme et al., 2021) and analyzed the relationship between the socio-economic, terrestrial, and hydrological factors on surface water quality via path analysis (Khan et al., 2021). The SCS-CN (soil conservation service curve number) model application was used to estimate runoff from small watersheds in different areas (Krisnayanti et al., 2021; Liu and Li, 2008). In addition to the above soil modeling methods, evaluation methods based on hierarchical analysis, frequency analysis, expert consultation, gray cluster analysis, and other indicator analysis methods are also widely used to measure soil conservation functions (Yang et al., 2016).

However, in the soil of larch plantations at different altitudes in northern Hebei Province (environmentally similar to our study site), the soil water conservation capacity at the higher altitude was higher than that in lower altitudes (Chen et al., 2012). Another study reports that in the forestland soil in the Lufeng area of Beijing, the soil water content increased with the altitude increase (Meng et al., 2016). The Wugong Mountain meadow in Jiangxi is a typical representative of the subtropical mountain meadow. With increasing altitude, meadow soil's effective state content of Fe, Cu, Zn, and B did not change significantly, and with the augmentation of the human disturbance, the effective state of Cu content increased (Deng et al., 2016, 2015). The total inorganic phosphorus content in meadow soil increased significantly with the altitude increase (Zhao et al., 2014). In addition, the soil available nitrogen content in the 0–20 cm soil layer of the meadow soil in this region was higher than that in the 20–40 cm soil layer (Yuan et al., 2015).

The meadow is a crucial habitat for many plants and animals (Halpern et al., 2012; McIlroy and Allen-Diaz, 2012; Viers et al., 2013). A popular scenic spot, Wugong Mountain offers landscape, ecological, and cultural diversity. Existing research, the research on Wugong Mountain mainly focuses on mountain meadows (Liu et al., 2018), geology (Li et al., 2018), soil quality (Li et al., 2017, 2020), effects of tourism (Rana et al., 2022) and ecological quality (Gao et al., 2022). In contrast, little is studied on the meadow soil's hydro-physical characteristics and water conservation function of Wugong Mountain. Therefore, evaluating the soil water conservation function of Wugong Mountain meadow at different altitudes is of great significance. This study clarifies the hydrological characteristics of meadows and helps realize the soil properties of certain altitudes and depths of Wugong Mountain. The results provide an in-depth understanding of soil hydrological characteristics, the theoretical basis for scientific research, ecological restoration, and sustainable management of subtropical mountain meadows.

## 2 Materials and methods

### 2.1 Overview of the research area

Wugong Mountain (114°10′–114°17′E, 27°25′–27°35′N) located in the southeast of Pingxiang City, Jiangxi Province, China. There are two rivers in this watershed: the Xiangjiang and the Ganjiang. The area covers about 970 km^2^, with a length of 120 km. The annual average temperature is 14–16 °C, the average annual humidity is 70–80%, and the average annual rainfall is 1350–1570 mm (Rana et al., 2022). Mostly granite and gneiss are found in Wugong mountain, and the peak is about 1918.3 m high (Yuan et al., 2015). There are large mountain meadow areas at altitudes of 1600 m and higher. The vegetation in this meadow soil is gramineous, including *Miscanthus sinensis*, *Arundinella anomala*, and *Perotis indica*. The region contains a small number of *Polygonaceae*, *Rosaceae*, *Labiatae*, and *Cruciferae*; however, *Miscanthus sinensis* is the most widely distributed of them (Cheng, 2014).

### 2.2 Test design and sample collection

The study was conducted in the year 2018. From the distribution edge area of the meadow to the peak, every 100 m (m), we set four altitude gradient treatments in 1600, 1700, 1800, and 1900 m. In each meadow area with no human interference to the altitude gradient, three 10 m × 10 m repeated samples were randomly set. The detail of different experimental treatments and the basic overview of the samples were shown (Table 1). Each sample area is about 10 m × 10 m, with three sample points, with every point spacing at about 5 m. The samples were divided into two layers of 0–20 cm and 20–40 cm of soil. Two circular knives were used for sampling in each layer (the circular knives were stainless, the upper and lower covers were aluminum, the specification was 50.46 mm × 50 mm, and the cubage was 100 cm^3^). The study was conducted according to the standard list of experiments and calculation methods (Institute of Forestry, 1999a, Institute of Forestry, 1999b), and combined with the relevant academic research results (He et al., 2015) was followed. The infiltration and drying methods were used to sample unit weight, capillary porosity, non-capillary porosity, total porosity, the mass water content of the soil, maximum water holding capacity, minimum water holding capacity, and capillary water holding capacity. The double-ring leakage method determined soil infiltration characteristics (He et al., 2015). The calculation formula for each indicator is as follows (Eqs. (1), (2), (3), (4), (5), (6), (7), and (8)):

(1) Soil physical index

(1) $$Pb=\frac{G}{V}$$

(2) $$Pn=0.1\times \left[W1-W2\right]\times \frac{Pb}{Pw}$$

(3) $$Pc=0.1\times W2\times \frac{Pb}{Pw}$$

(4) P=Pn+Pc

where: Pb = soil bulk density (g·cm^−3^), G = the quality of the dried soil inside the ring cutter (g), V = cutting ring size (cm^3^), Pn = non-capillary porosity (%), Pc = capillary porosity (%), W_1_ = maximum water holding capacity (g·kg^−1^), W_2_ = capillary moisture capacity (g·kg^−1^), Pw = density of water (kg·m^3^), P = total porosity (%).

**Table 1:** The geographical positions of the different treatments of mountain meadow.

Experimental treatments/m	Altitude of sample plots/m	Slope degree (°)	Slope direction	Longitude (E)	Latitude (N)	Main vegetation type	Vegetation coverage rate (%)
1600	1593	6	NE20°	114°10′31.50	27°27′57.76	*Miscanthus ​sinensis*	100
	1588	8	NE23°	114°10′35.24	27°27′57.02	*Miscanthus ​sinensis*	95
	1583	10	NE22°	114°10′37.05	27°27′26.76	*Miscanthus ​sinensis*	99
1700	1695	7	NE28°	114°10′25.55	27°27′43.38	*Miscanthus ​sinensis*	98
	1699	8	NE24°	114°10′27.55	27°27′46.62	*Miscanthus ​sinensis*	100
	1699	8	NE22°	114°10′28.05	27°27′48.94	*Miscanthus ​sinensis*	96
1800	1811	10	NE27°	114°10′25.72	27°27′29.43	*Miscanthus ​sinensis*	97
	1802	8	NE27°	114°10′25.75	27°27′30.79	*Miscanthus ​sinensis*	98
	1799	6	NE26°	114°10′24.70	27°27′31.03	*Miscanthus ​sinensis*	95
1900	1907	7	NE25°	114°10′26.09	27°27′16.76	*Miscanthus ​sinensis*	98
	1904	9	NE27°	114°10′25.16	27°27′20.22	*Miscanthus ​sinensis*	97
	1903	6	NE29°	114°10′24.79	27°27′20.60	*Miscanthus ​sinensis*	100

Where the soil bulk density is the dry bulk density (Zhou et al., 2016), which is equal to the soil density (Zhang et al., 2015); the density of water is 1000 kg m^3^.

(2) Soil water retention index

(5) $$W1=\frac{\left[G1-G2\right]}{G2}\times 1000$$

(6) $$W2=\frac{\left[G3-G1\right]}{G1}\times 1000$$

(7) $$W3=\frac{\left[G4-G1\right]}{G1}\times 1000$$

(8) $$W4=\frac{\left[G5-G1\right]}{G1}\times 1000$$

where: W_1_ = soil water content (g·kg^−1^), W_2_ = maximum water holding capacity (g·kg^−1^), W_3_ = capillary moisture capacity (g·kg^−1^), W_4_ = minimum water holding capacity (g·kg^−1^), G_1_ = the quality of the dried soil inside the ring cutter (g), G_2_ = mass of wet soil in-ring cutter (g), G_3_ = mass of wet soil in the ring cutter after 12 h infiltration (g), G_4_ = wet soil mass in-ring cutter after 2 h on dry sand (g), G_5_ = wet soil mass in-ring cutter after 72 h on dry sand (g).

(3) Soil permeability index
  o Initial infiltration rate = Infiltration volume/infiltration time during the initial infiltration period. The initial infiltration time in this study is 2 min.
  o Steady infiltration rate: the infiltration rate when the infiltration amount per unit time tends to be stable.
  o Average infiltration rate = total infiltration amount at steady infiltration/time at steady infiltration.
  o Since the permeation rate of all soil samples was stable before 60 min, in order to facilitate comparison, the total amount of permeation was taken within 60 min for convenience of comparison.

### 2.3 Statistical analysis

The differences in water conservation and physical properties of mountain meadow soil treated with different altitudes (α = 0.05) were analyzed using one-way analysis of variance (ANOVA), IBM SPSS Statistics 21 program. When the differences were significant, the Duncan method was used for multiple comparisons. The principal component analysis (PCA) and case ranking were used to compare the water conservation functions of soil at different altitudes and depths.

## 3 Results

### 3.1 Physical characteristics of soil in the mountain meadow at different altitudes

**Figure 1:**
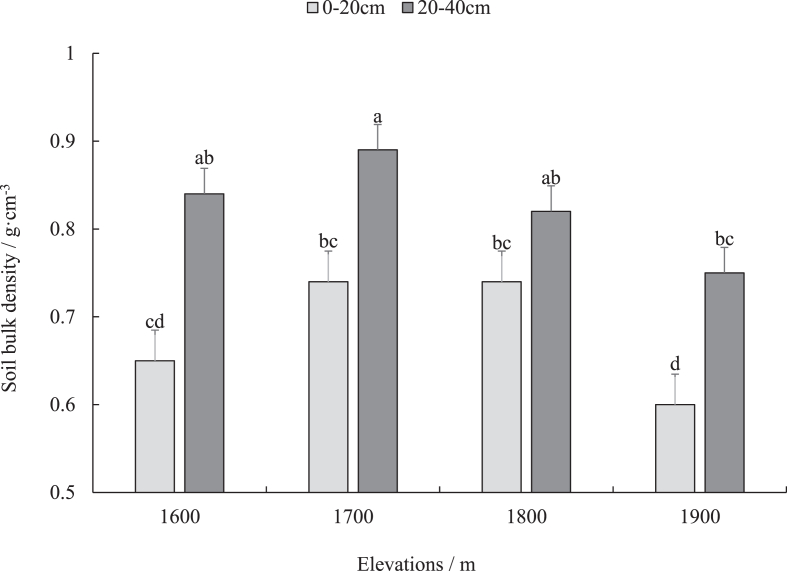
Soil bulk density of mountain meadows at different elevations. The lowercase letters represent significant differences (*P* < 0.05) between different elevations and soil depths.

Characteristics of bulk density distributions at different altitudes – the bulk density of the meadow soil at different altitudes distributed between 0.6–0.74 g cm^−3^ and 0.75–0.89 g cm^−3^ in the 0–20 cm and the 20–40 cm soil layer, respectively (Figure 1). In the altitudes of 1700–1900 m, the bulk density decreased with the increase of altitude gradient, and the bulk density of the meadow soil at 1600 m is slightly less than 1700 m. In the 0–20 cm soil layer, the altitudes of 1900 m differed significantly from the 1700 m–1800 m range (*P* < 0.05). The 20–40 cm soil layer shows a significant difference between 1900 m and 1700 m (*P* < 0.05). From the vertical direction, the bulk density of the upper soil layer at each altitude is lower than that of the lower soil layer, and there are significant differences between different soil layers at altitudes of 1600, 1700, and 1900 m (P < 0.05).

**Figure 2:**
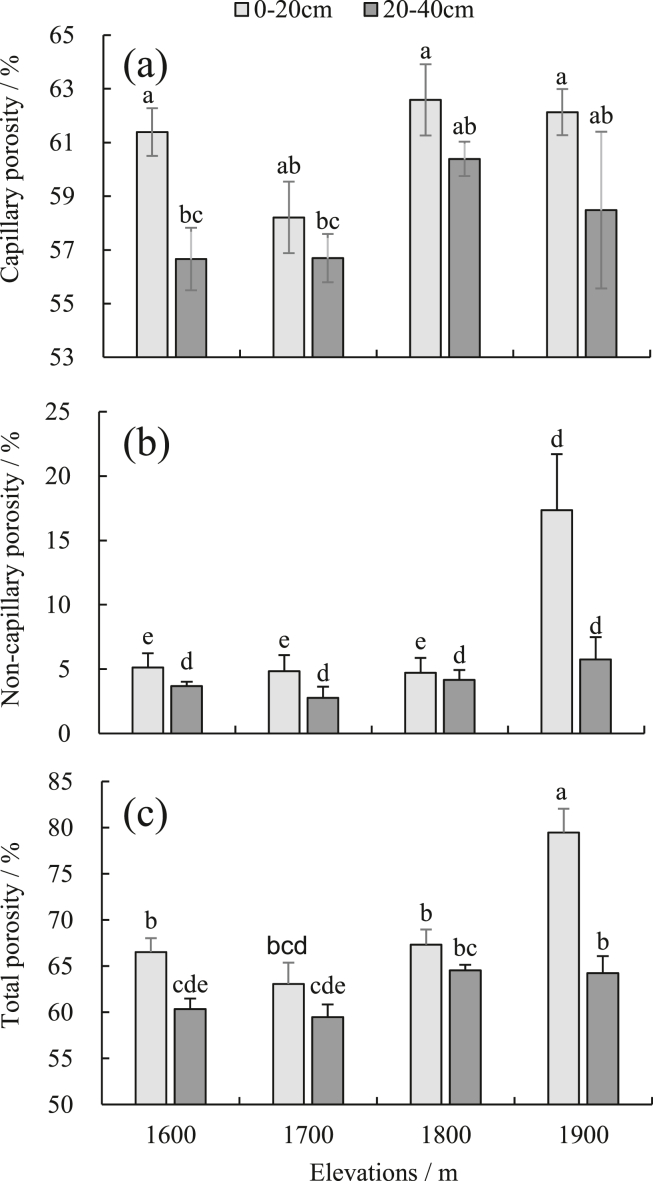
(a) Soil capillary porosity of mountain meadow at different elevations, (b) soil non-capillary porosity of mountain meadow at different elevations, (c) soil total porosity of mountain meadow at different elevations. The lowercase letters represent significant differences (*P* < 0.05) between different elevations and soil depths.

Characteristics of soil porosity distribution at different altitudes – The distribution of capillary porosity and non-capillary porosity in the soil at different altitudes is not obvious, but the total porosity increases with the increase of altitudes (Figure 2a, b, c). The altitude range of 1600 m is slightly greater than 1700 m, showing that the effect of altitude on soil porosity in the mountainous meadow is reflected in total porosity. At 0–20 cm, soil capillary porosity ranges from 58.21% to 62.59% at different altitudes (*P >* 0.05). The distribution ranges of non-capillary porosity and total porosity are 4.73%–17.35% and 63.06%–75.83%, respectively. The 1900 m differed significantly from other altitudes (*P <* 0.05); at 20–40 cm, the distribution ranges of capillary porosity and non-capillary porosity were 56.66%–62.13% and 2.76%–5.75%, respectively, and the difference was not significant. The distribution range of total porosity is 59.47%–67.88% (*P* > 0.05), and the altitude of 1900 m differed significantly from the 1700 and 1600 m range (*P* < 0.05). From the vertical direction, 0–20 cm soil capillary porosity and non-capillary porosity and total porosity were greater than the 20–40 cm soil layer. The difference in capillary porosity in different soil layers is significant at 1600 m altitude (*P* < 0.05), the non-capillary porosity was significant at 1600, 1700, and 1800 m altitude (*P* < 0.05), and the total porosity is significant in the altitudes of 1600, 1700, and 1900 m (*P* < 0.05).

### 3.2 Soil water characteristics of meadowland at different altitudes

**Figure 3:**
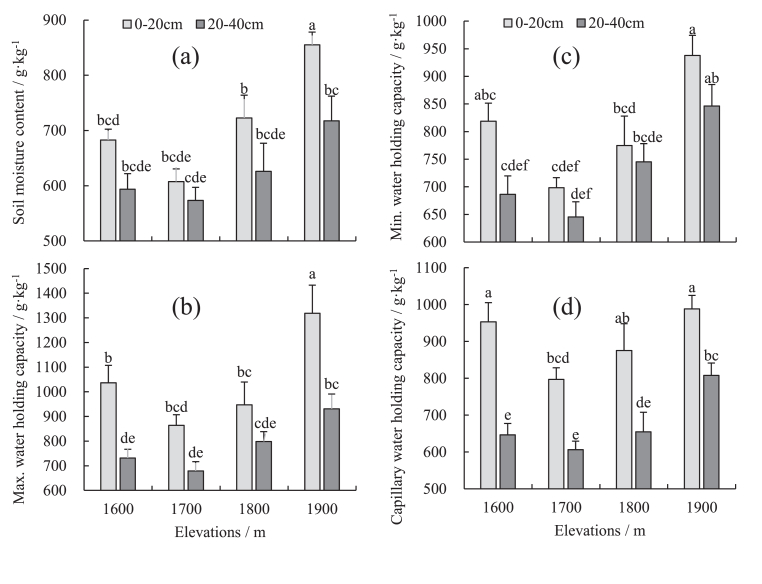
(A) Soil moisture content of mountain meadow at different elevations, (b) soil maximum water holding capacity of mountain meadow at different elevations, (c) soil minimum water holding capacity of mountain meadow at different elevations, (d) soil capillary water holding capacity of mountain meadow at different elevations. The lowercase letters represent significant differences (*P* < 0.05) between different elevations and soil depths.

The distribution of soil water content and water holding capacity at different altitudes of Wugong mountain is similar. The overall performance is that the water content or water holding capacity increases with the altitude, and the altitude of 1600 m is slightly greater than 1700 or 1800 m (Figure 3a, b, c, d). In the 0–20 cm soil layer, the soil water content distribution ranged is 607.51–855 g kg^−1^ at 1900 m, significantly differing from other altitudes (*P* < 0.05). The maximum soil water capacity distribution range is 863.82–1318.87 g kg^−1^ at 1900 m, which is also significantly different from other altitudes (*P <* 0.05), and the soil capillary water capacity is 796.85–988.39 g kg^−1^. The altitude range of 1700 m differed significantly from that of 1900 m and 1600 m (*P* < 0.05), and the soil minimum water capacity distribution range is 698.53–937.75 g kg^−1^. The altitude range of 1900 m differed significantly from that of 1800 m and 1700 m (*P* < 0.05).

In the 20–40 cm soil layer, the soil water content distribution range is 625.96–717.66 g·kg^−1^, and the difference between altitudes is significant (*P* > 0.05). The distribution range of the maximum water holding capacity of the soil is 678.86–930.57 g kg^−1^. Besides, the altitude of 1900 m has differed significantly from that of 1600 m and 1700 m altitudes (*P* < 0.05). The capillary soil water holding capacity distribution range is 606.05–807.6 g kg^−1^, and the 1900 m has differed significantly from the other three altitudes (*P* < 0.05). The minimum soil water holding capacity distribution range is 645.29–846.44 g kg^−1^, and the altitude range of 1900 m differed significantly from 1800 m and 1700 m (*P* < 0.05).

From a vertical direction, the water content and water holding capacity of the 0–20 cm soil layer are greater than that of the 20–40 cm soil layer. The soil water content of different soil layers significantly differs at the 1900 m altitude (*P* < 0.05). The maximum soil water holding capacity is significantly different between 1600 m and 1900 m altitudes (*P* < 0.05), and the capillary soil water holding capacity is significantly different in the four altitudes of 1600 m–1900 m (*P* < 0.05). There is no significant difference in the minimum soil water holding capacity at each altitude (*P* > 0.05).

### 3.3 Soil permeability in different altitude mountain meadows

Soil permeability is one of the important indexes of water conservation capacity, which is affected by soil porosity, bulk density, soil viscosity, and other factors (Zhang et al., 2015). The soil infiltration rate of mountain meadows at different altitudes; Initial infiltration rate >average infiltration rate > steady infiltration rate shown (Table 2). In the 0–20 cm soil layer, the initial infiltration rate, steady infiltration rate, and average infiltration rate decreased with the altitude from 1600 m to 1800 m. It increases again at 1900 m, and the distribution range of initial permeability is 3.42–30.62 mm min^−1^. There is a significant difference between the altitudes of 1600 m and 1800 m (*P* < 0.05). The steady infiltration rate distribution range is 2.46–26.27 mm min^−1^, and there is a significant difference between 1600 m and 1800 m at 1900 altitudes (*P* < 0.05). The average infiltration rate is 2.85–27.41 mm min^−1^, and there is a significant difference between the altitudes of 1600 m and 1800 m (*P* < 0.05).

**Table 2:** The soil infiltration rate of mountain meadows at different altitudes. The lowercase letters indicate the significance level of the values.

Soil depth/cm	Altitude/m	Initial infiltration rate/mm·min^−1^	Steady infiltration rate/mm·min^−1^	Average infiltration rate/mm·min^−1^
0–20	1600	30.62 ± 7.07^d^	26.27 ± 4.73^c^	27.41 ± 5.24^d^
	1700	14.26 ± 5.78^bc^	12.26 ± 5.02^b^	13.19 ± 5.25^c^
	1800	3.42 ± 1.45^abc^	2.46 ± 1.00^a^	2.85 ± 1.09a^b^
	1900	15.91 ± 7.07^c^	7.82 ± 3.59^ab^	10.64 ± 4.88^bc^
20–40	1600	2.62 ± 1.29^ab^	1.82 ± 1.06^a^	2.15 ± 1.20^ab^
	1700	9.85 ± 4.54^abc^	8.24 ± 3.82^ab^	8.83 ± 4.09^abc^
	1800	0.15 ± 0.05^a^	0.11 ± 0.04^a^	0.12 ± 0.03^a^
	1900	7.13 ± 4.19^abc^	2.34 ± 1.59^a^	3.49 ± 1.86^abc^

In the 20–40 cm soil layer, there is no obvious change with altitude. The initial infiltration rate distribution range is 0.15–9.85 mm min^−1^, and the altitudes of 1700 m, 1600 m, and 1800 m were significantly different (*P* < 0.05). The stable infiltration rate distribution range is 0.11–8.24 mm min^−1^, and the 1800 m differs significantly from other altitudes (*P* < 0.05). The average infiltration rate is 0.12–8.83 mm min^−1^, and there is a significant difference between 1700 and 1600 m at 1800 altitudes (*P* < 0.05). From the vertical direction, the soil infiltration rate of the 0–20 cm soil layer is higher than that of the 20–40 cm soil layer, and the initial infiltration rate, stable infiltration rate, and average infiltration rate of different soil layers were significantly different at 1600 m altitude (*P* < 0.05).

### 3.4 Comprehensive evaluation of soil water conservation function in the meadow at different altitudes

The principal component analysis was carried out with Soil water content (*A*_*1*_), maximum water holding capacity (*A*_*2*_), minimum water holding capacity (*A*_*3*_), capillary water holding capacity (*A*_*4*_), bulk density (*A*_*5*_), capillary porosity (*A*_*6*_), non-capillary porosity (*A*_*7*_), total porosity (*A*_*8*_), initial infiltration rate (*A*_*9*_), stable permeability (*A*_*10*_), average infiltration rate (*A*_*11*_). As the evaluation index, the water conservation functions of soil at different altitudes and depths of different soil layers were comprehensively compared. The eigenvalues of the first three principal components are 5.993, 3.075, and 1.652, respectively, and the cumulative contribution rate is 97.455%, which meets the conditions for explaining the population variance and has relatively small information loss (Table 3).

**Table 3:** Principal component analysis of soil water conservation function in the mountain meadow.

Parameter	Principal component		
	P_1_	P_2_	P_3_
*A*_*1*_	0.893	−0.279	0.323
*A*_*2*_	0.947	−0.162	−0.270
*A*_*3*_	0.929	−0.065	0.321
*A*_*4*_	0.945	0.155	0.247
*A*_*5*_	−0.959	0.023	0.083
*A*_*6*_	0.128	0.315	0.928
*A*_*7*_	0.721	−0.321	−0.599
*A*_*8*_	0.951	−0.160	−0.058
*A*_*9*_	0.319	0.929	−0.145
*A*_*10*_	0.173	0.965	−0.181
*A*_*11*_	0.226	0.959	−0.164
Characteristic root	5.993	3.075	1.652
Variance contribution rate (%)	54.480	27.957	15.019
Cumulative contribution rate (%)	54.480	82.436	97.455

The first principal component has a significant correlation with soil water content (*A*_*1*_), maximum water holding capacity (*A*_*2*_), minimum water holding capacity (*A*_*3*_), capillary water holding capacity (*A*_*4*_), bulk density (*A*_*5*_), non-capillary porosity (*A*_*7*_), total porosity (*A*_*8*_). The second principal component has a significant correlation with the initial infiltration rate (*A*_*9*_), stable permeability (*A*_*10*_), and average infiltration rate (*A*_*11*_). The third principal component was closely related to capillary porosity (*A*_*6*_), according to the factor score coefficient matrix (Table 4). The principal component equation of each principal component is as follows (Eqs. (9), (10), and (11)):

(9) f1=0.149A1+0.158A2+0.155A3+0.158A4−0.16A5+0.021A6+0.12A7+0.159A8+0.053A9+0.029A10+0.038A11

(10) f2=−0.091A1−0.053A2−0.021A3−0.05A4+0.007A5+0.102A6−0.104A7−0.052A8+0.302A9+0.314A10+0.312A11

(11) $$f_{3\;}＝\;0.195A_{1}-0.164A2+-0.194A3+0.149A4+0.05A5+0.562A6-0.363A7-0.035A8-0.088A9-0.11A10-0.099A11$$

*A*_*i*_ is the standardized data of each index.

**Table 4:** Soil water conservation function factor score coefficient matrix of mountain meadow.

Parameter	Principal component		
	P_1_	P_2_	P_3_
*A*_*1*_	0.149	−0.091	0.195
*A*_*2*_	0.158	−0.053	−0.164
*A*_*3*_	0.155	−0.021	0.194
*A*_*4*_	0.158	0.050	0.149
*A*_*5*_	−0.160	0.007	0.050
*A*_*6*_	0.021	0.102	0.562
*A*_*7*_	0.120	−0.104	−0.363
*A*_*8*_	0.159	−0.052	−0.035
*A*_*9*_	0.053	0.302	−0.088
*A*_*10*_	0.029	0.314	−0.110
*A*_*11*_	0.038	0.312	−0.099

According to the first (1), second (2), and third (3) principal component equations, the scores of water conservation function of upper (0–20 cm) and lower (20–40 cm) soil layers at different altitudes were calculated and ranked (Table 5). The comparison shows differences in the water conservation capacity of the upper and lower soil layers at the same altitude. The order of water conservation capacity in 0–20 cm soil layer is 1900 > 1600 > 1800 > 1700 m. Contrary, the order of water conservation capacity of 20–40 cm soil layer is 1900 > 1800 > 1700 > 1600 m. From the comprehensive average score, the order of soil water conservation capacity at different altitudes is 1900 > 1600 > 1800 > 1700 m.

**Table 5:** The comprehensive evaluation of soil water conservation function of mountain meadows at different altitudes.

Altitude/m	0–20 cm		20–40 cm		Average score	Order
	Score	Order	Score	Order		
1600	0.46	2	−0.75	4	−0.14	2
1700	−0.79	4	−0.43	3	−0.61	4
1800	−0.52	3	−0.09	2	−0.30	3
1900	0.84	1	1.27	1	1.06	1

## 4 Discussion

In this study, the hydrological and physical characteristics of meadow soil in the typical subtropical mountainous area of Wugong Mountain were systematically analyzed, and the soil-water conservation function of meadows in different altitudes was comprehensively evaluated. Results found that the altitude factor significantly affects mountain meadow soil-physical properties and water conservation capacity (P < 0.05), but the changes in soil hydro-physical indexes are not completely consistent with the change law of altitude gradient. In the vertical direction, the hydro-physical characteristics of different soil layers and the distribution of water conservation capacity are relatively uniform.

Soil water content is the proportion of soil liquid phase mass to solid-phase Mass, which reflects the soil water condition under the natural state, which directly affects vegetation growth and soil nutrient conversion, and has a control effect on rainfall infiltration and runoff stroke (Chen et al., 2012). The maximum water holding capacity of the soil is the maximum amount of water that can mainly maintain after the soil pores are filled. Soil capillary water holding capacity is mainly based on capillary gravity to overcome gravity and preserve capillary pores. It has the characteristics of fast-moving speed and large quantity, which is the dominant form of plant using soil water. The capillary water holding capacity comprises two parts: the capillary suspended water and the rising capillary water. The capillary suspended water is the soil water that is held in the soil capillary because of the capillary gravitation during the transportation process, which is the minimum water holding capacity of the soil, also known as the field water holding capacity, which mainly reflects the upper limit of the available water in the soil under the dry land state (Bai et al., 2016; Zhang and Zhou, 2015).

### 4.1 Altitude and soil water conservation capacity

In the altitudes of 1700–1900 m, soil bulk density decreases with altitude, while soil porosity, water holding capacity, and comprehensive water conservation function increase with altitude. At the altitude of 1600 m, the bulk density of soil is slightly lower than 1700 m, and other hydro-physical indexes and water conservation capacity are slightly higher than 1700 m or 1800 m but less than 1900 m. The distribution characteristics are similar to the research results (Chen et al., 2012; Lu et al., 2014; Wei et al., 2014). Soil bulk density is an essential reflection of the resistance of root distribution, soil permeability, and aeration. However, soil porosity is an essential channel for transmitting soil microorganisms, air, water, and nutrients. The soil water holding capacity and water content are essential indicators reflecting the water conservation capacity (Liu et al., 2006). Soil bulk density affects soil porosity, water permeability, and water storage capacity, and several indexes interact with each other. Soil water conservation capacity is a comprehensive reflection of all hydro-physical indices (Gao et al., 2016; Zhang et al., 2015). The altitude of the meadow distribution area is along with the 1700 m–1900 m altitudes.

On the one hand, the environment temperature decreases, the soil microbe's decomposition ability decreases, and soil organic matter accumulation increases. Therefore, reduce soil bulk density, and soil porosity increases. On the other hand, the vegetation was gradually reduced, including the water demand of the soil, and in contrast, the accumulation of water in the soil was higher. Thus, soil water content and water holding capacity gradually increased with increasing altitude. But, in the region of the 1600 m altitude, because on the brink of meadow distribution initial scope. There may be a certain edge effect, and the low altitude area of hydrothermal conditions advantage the vegetation growth momentum was good. The vegetation root system developed, and the higher the nutrient return. Therefore, the soil bulk density was slightly smaller than 1700 m above the sea level range, and other hydro-physical indexes, such as higher water conservation capacity than 1700 m or 1800 m. A similar study reported that (Li et al., 2012) the live root/soil volume ratio of vegetation was significantly and positively correlated with soil water holding capacity and porosity with an increase in the soil/root ratio and the adsorption capacity of live roots of herbage to water was the main factor affecting the water conservation of alpine meadow grass.

During the investigation of this area, there have been many instances of rainfall in the high-altitude area of Wugong Mountain, but no rainfall at low altitudes. Therefore, to adapt to this natural environment, the root structure of vegetation at high altitudes may be unique, and a single root's volume or surface area may be more extensive. The interaction between root characteristics and soil may also be one reason for the better soil hydro-physical characteristics and water conservation capacity in the 1900 m altitudes. There is potential for further exploration of this content in the future.

### 4.2 Hydro-physical characteristics in soil layers

The average thickness of the soil layer of the Wugong Mountain meadow is about 50 cm. The root distribution of the meadow is mainly 0–20 cm, and the humus layer on the surface of the soil layer is relatively thin. Therefore, this study sets the survey thickness of the meadow soil layer at 0–40 cm. The soil bulk density of 0–20 cm at each altitude is less than 20–40 cm, and soil porosity, water holding capacity, and water conservation capacity are higher at the upper level than at the lower level. As the soil layer deepens and vegetation roots decrease, the subsoil becomes more compacted, while the surface soil is relatively loose. Therefore, the bulk density of the subsoil is greater than that of the upper soil. In contrast, other upper soil's hydro-physical characteristics and water conservation capacity are more significant than the lower soil (Lu et al., 2014).

As a typical representative of the subtropical meadow ecosystem in southern China, it should carry the more comprehensive scientific research to improve the regional environment. While maintaining the largest “Cloud Grassland” landscape in southern China, how to protect better and use this kind of vegetation ecosystem can be the leading research content in the future.

## 5 Conclusion

The study analyzed the hydro-physical characteristics and the soil properties in meadow soil at 1600 m–1900 m altitude and the depth of 0–20 cm and 20–40 cm of soil layers. A systematic study in this area (Wugong Mountain meadow) was crucial to understanding the mountain meadow's soil and water conservation function at different altitudes for ecological restoration and sustainable management. Altitude significantly impacts the soil hydro-physical characteristics and water conservation function of Wugong Mountain meadows (*P* < 0.05), but the changing trends of various indicators are not entirely consistent with the altitude changes. We conclude that the comprehensive values of soil bulk density, porosity, and water holding capacity of this meadow soil have differed in the different altitudes and soil layers. In addition, the comprehensive study of soil water conservation function demonstrated that the soil water conservation capacity differs by the depth of the soil layer at altitude change. This research showed the altitude sequence of higher soil water conservation capacity at 1900, 1600, 1800, and 1700 m altitudes. Further study should be considered extensively, including the parameters and the large (altitude and soil layers) area coverage.

## Declarations

### Author contribution statement

Sohel Rana; Zhi Li: Conceived and designed the experiments, Analyzed and interpreted the data and Wrote the paper.

Xuna Cheng: Conceived and designed the experiments, Performed the experiments and Wrote the paper.

Zhen Liu; Yanmei Wang; Qifei Cai; Xiaodong Geng and Xiaomin Guo: Conceived and designed the experiments and Contributed reagents, materials, analysis tools or data.

Yanfang Wu; Chuanwei Hu; Razia Sultana Jemim: Performed the experiments and Analyzed and interpreted the data.

### Funding statement

This work was supported by Henan Provincial Postdoctoral Science Foundation of China [202002053], National Natural Science Foundation of China [31360177], Key Forestry Science and Technology Promotion Project of China Central Government [GTH[2020]17].

### Data availability statement

Data will be made available on request.

### Declaration of interest's statement

The authors declare no conflict of interest.

### Additional information

No additional information is available for this paper.
